# Ischemic and Hemorrhagic Cerebrovascular Events Related to COVID-19 Coagulopathy and Hypoxemia

**DOI:** 10.3390/ijerph191811823

**Published:** 2022-09-19

**Authors:** Michał Sojka, Anna Drelich-Zbroja, Maryla Kuczyńska, Mateusz Cheda, Izabela Dąbrowska, Ewa Kopyto, Izabela Halczuk, Monika Zbroja, Weronika Cyranka, Tomasz Jargiełło

**Affiliations:** 1 Department of Interventional Radiology and Neuroradiology, Medical University of Lublin, 20-090 Lublin, Poland; 2 Students’ Scientific Society at the Department of Interventional Radiology and Neuroradiology, Medical University of Lublin, 20-090 Lublin, Poland

**Keywords:** COVID-19, ischemic stroke, hypoxia, hemorrhagic stroke, hypoxic-ischemic encephalopathy

## Abstract

Since the very beginning of the COVID-19 pandemic, numerous researchers have made an effort to determine the molecular composition of the SARS-CoV-2 virus, and the exact pathomechanism through which the virus exerts such a devastating effect on the host/infected organism. Recent scientific evidence highlights the affinity of the virus towards ACE2 receptors, which are widespread in multiple human systems, including the central nervous system (CNS) and cerebral vessels. Such an affinity may explain endothelial dysfunction and damage that is observed in COVID-positive patients in histopathological studies, with subsequent dysregulation of the cerebral circulation leading to transient or acute cerebrovascular accidents. In this paper, we aimed to evaluate the effects of COVID-related hypoxemia and direct viral invasion on the cerebral circulation, with special respect to the postulated pathomechanism, vulnerable groups of patients, clinical course and outcomes, as well as diagnostic imaging findings.

## 1. Introduction

In December 2019, the first known COVID-19 (Coronavirus Disease 2019) case was been identified, only one month after (January 2020) the World Health Organization (WHO) considered it a public health emergency, subsequently qualifying it as a pandemic on 11 March 2020. Coronavirus 2 (SARS-CoV-2) has been identified as the primary source of infection causing COVID-19, resulting in severe acute respiratory syndrome. Since 2021, multiple variants of this virus have emerged and become prevalent across many countries. As of 5 December 2021, there have been more than 265 million affected cases and 5.25 million confirmed deaths, making the pandemic among the most lethal in history [1].

Along with leading to significant social and economic disruption worldwide, the pandemic was and continues to be a serious public health burden. While countering a pandemic is immensely exacting, it is also necessary to consider the patients with chronic and acute illnesses, such as stroke and myocardial ischemia, when confronting this challenge. The research regarding the impact of the SARS-CoV-2 infection on human health and its interaction with already known health issues is constantly ongoing [2].

The course of the COVID-19 virus disease can be both asymptomatic and life-threatening. Among the symptoms of the infection being described are cardiac damage, kidney injuries, coagulopathy and neurological symptoms including headache and dizziness, cranial nerves damage such as anosmia, cerebrovascular diseases, and encephalopathies. Up to 36% of patients with COVID-19 have been observed to have neurological symptoms [3].

Since 2019 and the announcement of the first strain of the novel coronavirus SARS-CoV-2, it became clear that COVID-19 has become a nationwide problem, and a burden for global healthcare systems and socioeconomics. Until now, we have faced subsequent pandemic waves evoked by different viral variants or mutations exhibiting various affinity towards particular body systems, leading to more or less pronounced symptoms. Although novel coronavirus disease (COVID-19) may be asymptomatic, critics should be aware of possible severe vascular neurologic complications of the infection. Previous reports were full of considerations regarding the contribution of traditional vascular risk factors, outcomes of COVID-positive patients compared to historical cohorts, and possible causative factors, including embolic and cryptogenic scenarios. However, the majority of investigations were based on small populations of patients, leaving many unknowns and contradictory data. While SARS-CoV-2 infection seems to be associated with general hypercoagulability, either due to systemic inflammatory response and cytokine storm or ACE-2-mediated endothelial dysfunction, recent reports indicate the presence of circulating antibodies against Platelet Factor 4 (PF4) in some COVID-positive individuals, which drive systemic response resembling the Heparin-Induced Thrombocytopenia and leading to hemorrhagic events. As the pathophysiology of COVID-19 vascular neurologic complication seems to be more complex than first thought, comprehensive literature reports including some general explanations regarding mechanistic aspects of the infection, clinical and diagnostic profiles of the affected patients, along with prognostic factors, and expected outcomes seem to be a valuable adjunct to clinical practice.

In order to facilitate successful management of the affected patients, it is imperative to recognize such COVID-19-related clinical patterns and present them to the global audience, thereby preventing the economic and social consequences of coronavirus-related disability, a concern for the global public health sector.

A six-section structure has been used to facilitate reading:

- In Section 2, we discuss possible molecular mechanisms contributing to both ischemic and hemorrhagic neurological complications of COVID-19, including theories regarding ACE-2 receptors, cytokine storms, and Weibel–Palade bodies;
- Section 3 introduces hypoxic-ischemic encephalopathy, emphasizing its origins among COVID-19 patients (prolonged hypoxemia, acute respiratory distress syndrome, cardiac complications, ventilatory therapy, and sedation), distinguishing vulnerable patients, possible clinical scenarios and outcomes, and presenting the major imaging findings;
- Section 4 provides a comprehensive consideration of ischemic complications caused by SARS-CoV-2 infection, including relevant definitions, and special attention to epidemiology, risk factors, and diagnostic imaging;
- In Section 5, we address similar epidemiological and clinical problems associated with hemorrhagic stroke in COVID-19 patients;
- Regarding possible limitations of the study, Section 6 provides general conclusions concerning COVID-19 major cerebrovascular complications.

## 2. Potential Mechanisms of Both Hemorrhagic and Ischemic Stroke

The commonly described potential mechanism for the induction of cerebrovascular disease is based on the affinity of SARS-CoV-2 to ACE2 receptors. The virus enters using the S-glycoprotein and binds to ACE2 receptors, resulting in damage to intracranial arteries, and causing rupture of the vessel wall [4,5,6,7]. Along with systemic inflammation, the cytokine storm plays a role not only in the direct induction of stroke, but may also act indirectly, by generating other systemic complications that predispose to cerebrovascular events. Following the binding of SARS-CoV-2 to the ACE2 receptor and interaction with transmembrane serine protease-2 (TMPRSS2), serum angiotensin II levels are increased. This process results in a massive increase in inflammatory cytokines, including interleukin-1beta (IL-1-beta), interferon-gamma, interferon-induced protein 10, monocyte chemoattractant protein 1, as well as IL-1, IL-2, IL-6, and tumor necrosis factor-alpha (TNF-alpha). It not only leads to endothelial dysfunction, but may evoke cardiovascular effects, such as reduced coronary blood flow, direct heart damage, microthrombosis, and atherosclerotic plaque destabilization as well. In fact, these systemic COVID-19 complications may indirectly aid the occurrence of both ischemic and hemorrhagic stroke [4,5,6,7,8,9]. The pro-inflammatory cytokines as described above, together with monocyte-derived macrophages (MDMs) activated by SARS-CoV-2 invasion, can induce tissue factor (TF) expression by endothelial cells. As a matter of fact, MDMs can also express tissue factor themselves. In turn, TF initiates the extrinsic coagulation pathway, leading to fibrin elevation and blood clotting. Such a constellation of events may speak in favor of a possible correlation between ischemic stroke and COVID-19 [4,10,11]. There are several studies that link the above pro-inflammatory cytokines with hypercoagulable states due to their role in releasing factor VIII, D-dimers, fibrinogen, and von Willebrand factor (VWF) [12,13]. It is specifically the VWF exocytosis from Weibel–Palade bodies in response to increased levels of IL-6, IL-8 and TNF-α, which is linked with acute inflammatory and viral conditions [14]. The increased levels of circulating VWF, coupled with downregulation of the ADAMTS13 protease (metalloprotease responsible for the degradation of large VWF multimers), can induce a prothrombotic microangiopathy-like process [13,14,15,16]. It is possible that the imbalance between VWF levels and ADAMTS13 contributes to the thrombosis caused by COVID-19 [14]. According to some small series studies, the increase in VWF activity in COVID-19 patients was found to be 600–800% of the normal range [14,15,17]. Furthermore, studies have implicated VWF/ADAMTS13 imbalance as a risk factor for ischemic stroke [14,18], and suggested that a similar mechanism can underlie stroke in other viral infections [14,19].

A cytokine storm and endothelial damage can also lead to extrusion of the Weibel–Palade bodies, resulting in increased levels of angiopoietin-2 [16,20]. Together with angiopoietin-1 and Tie2 (tyrosine-protein kinase receptor), angiopoietin-2 is crucial for both physiologic angiogenesis and the formation of pathologic blood vessels [20]. Angiopoietin-1 and angiopoietin-2 are antagonistic ligands that bind with a similar affinity to Tie2 [20]. In healthy individuals, Tie2 maintains endothelial homeostasis and promotes the expression of the anticoagulant genes, as it remains activated due to constant angiopoietin-1 secretion from both the perivascular cells and blood platelets [16]. In the COVID-19 infection, the rapid increase in angiopoietin-2 concentrations results in direct activation of Tie2, setting off a chain reaction of neoangiogenesis and inflammation (which later transforms into a vicious cycle), leading to hypercoagulability and the emergence of defective capillaries prone to microthrombosis [16,20]. Considering the above, angioprotein-2 might be a useful marker for endothelial dysfunction in COVID-19 patients, and a relevant predictive factor for direct admissions to the ICU [21].

As for intracranial hemorrhage, there are several proposed mechanisms that link endothelial impairment following ICH with COVID-19 infection. Most notably is the aforementioned cytokine storm and activation of the coagulation cascade, leading to various thrombotic events caused by microvascular thrombosis [22,23,24]. Certain authors suggest that COVID-19 infection may disrupt the blood–brain barrier in numerous ways (e.g., by destabilizing tight junction proteins), resulting in ICH. Moreover, several studies strongly suggest the involvement of the renin–angiotensin system (RAS) in the pathogenesis of both ICH and COVID-19 [22,23,24]. A decrease in ACE2 expression during SARS-CoV-2 infection results from the virus binding to ACE2 receptors [4,5,6,7]. Subsequently, increased serum levels of angiotensin 2 result in overactivation of the classical RAS pathway, along with a decrease in activation of the cerebral alternative RAS axis, leading to cerebral vascular dysregulation [22,25,26]. Such a process is likely to impair endothelial function and contribute to blood pressure deregulation, which may also explain the correlation between hypertension and hemorrhagic stroke [4,6,27,28]. In addition, COVID-19 may be associated with consumption coagulopathy due to fibrinogen depletion, which increases the risk of ICH [4,29,30].

## 3. Hypoxic-Ischemic Encephalopathy

### 3.1. Prevalence, Clinical Presentation, and Neuropathology

Hypoxic-ischemic encephalopathy (HIE), also referred to as hypoxic/anoxic brain injury, is defined as a diffuse cerebral insult due to oxygen deprivation resulting from systemic conditions, including prolonged or severe hypoxemia, hypotension (mean arterial blood pressure below 65 mm Hg) or cardiac arrest, and leading to new cerebral dysfunction or characteristic findings on cross-sectional imaging of the brain, i.e., computed tomography or magnetic resonance [31,32].

In a prospective multicenter observational study on 4491 consecutive patients with laboratory-confirmed infection with SARS-CoV-2 (RT-PCR SARS-CoV-2–positive patients) [31], there was an observed development of new neurological symptoms in 606 (over 13%) of the study subjects in a median of 2 days from COVID-19 onset [33]. Hypoxic/ischemic injury was the fourth most common neurological manifestation (after toxic/metabolic encephalopathy—6.8%, stroke—1.9%, and seizure—1.6%), affecting 1.4% of the study population, and accounting for 11% (65/606) of all neurological symptoms. Apparently, patients with COVID-19 and neurologic disorders were exposed to an increased risk of in-hospital mortality (HR 1.38, 95% CI 1.17–1.62, *p* < 0.001) and less likely to be discharged home (HR 0.72, 95% CI 0.63–0.85, *p* < 0.001). The unique aspect of the study was the rigorous approach to the identification of specific neurological diagnoses, including the evaluation of each patient by neurologists and the use of accepted diagnostic criteria (not solely clinical suspicion) [33]. Frontera et al. suggested that patients with HIE generally presented with higher markers of severe illness, including the SOFA score, and a necessity for ICU admission or intubation. Most of them presented with severe or protracted hypoxemia (88%). In comparison to other TME etiologies, this group exhibited lower median oxygen saturation levels (80% vs. 88%, *p* < 0.001), higher median number of desaturation episodes (5 vs. 1, *p* < 0.001), and more often was hypotensive (median MAP of 55 mm Hg vs. 67 mm Hg, *p* < 0.001) [21].

The hypoxic-ischemic injury was observed both in patients after cardiac arrest who developed new CNS deficits, as well as individuals with prolonged/severe hypoxia or hypotension with new neurological symptoms and typical findings on diagnostic imaging [33].

In the same cohort of 4491 patients with COVID-19, 559 were diagnosed with toxic metabolic encephalopathy (TME), with hypoxic-ischemic encephalopathy being the second most frequent etiology (59%). Multiple etiologies were reported in nearly 80% of patients [31]. Patients with toxic-metabolic injury were generally older, more often had dementia and required intubation (all *p* < 0.001). Additionally, longer hospital stays were observed in this group of patients [31].

Despite initial historical considerations, recent data support the deleterious effects of isolated hypoxemia, even if the episodes were brief. Such observations have been well documented in hikers at altitude repeatedly exposed to hypoxemia (oxygen saturation < 65%), in whom transient cognitive deficits were observed, along with abnormalities in both primary and secondary motor cortex shown on MRI. The effects were even more pronounced among patients diagnosed with acute respiratory distress syndrome (ARDS). Long-term cognitive deficits following severe hypoxemia were observed in 30% to 55% of patients in this group [31].

Increasing numbers of ARDS cases were also observed during the COVID-19 pandemic due to the use of rescue therapy, such as extracorporeal membrane oxygenation (ECMO) support. These were also related to the occurrence of new neurological deficits, including ischemic stroke (1.1%), intracranial hemorrhage (5.9%), and hypoxic-ischemic brain injury (0.3%) [34].

The direct link between COVID-19 and neuropathological findings remains nothing less than controversial. Autopsy studies performed by Thakur et al. on 41 consecutive patients with COVID-19 confirmed the presence of SARS-CoV-2 RNA in the majority of the investigated brain specimens; however, the viral load measured by qRT-PCR was either low or very low, especially compared to the nasal epithelia. The authors failed to detect specific SARS-CoV-2 proteins using immunohistochemistry [35].

An additional systematic review (by Mukerji and Salomon) indicates that autopsy records of COVID-19 patients with clinically apparent cognitive impairment, in general, have not identified neural changes or substantial inflammation, which are commonly associated with viral infections. In addition, viral genetic material was minimal or absent [36].

Cosentino et al. performed a systematic review of 45 articles describing neuropathological findings in a total of 438 COVID-19 patients. Hypoxic-ischemic lesions were detected in nearly 41% of the study population, following micro- and astrogliosis (52.5% and 45.6%, respectively), and inflammatory infiltrates (44.0%). No significant correlation was observed between the incidence of hypoxemic-ischemic lesions in neuropathological specimens, and neither viral RNA load (*p* = 0.951) nor specific proteins (*p* = 0.705). However, an association was found with the presence of cardiovascular risk factors (*p* < 0.001). Hypoxic-ischemic lesions (53 of 138 patients, 48.6%; *p* < 0.001), along with brain edema and inflammation, were more frequently associated with neurological symptoms; nonetheless, only two individuals with confirmed hypoxemic-ischemic lesions developed clinically apparent hypoxic encephalitis. These findings would, rather, advocate for the relevance of hypoxemia and inflammation in the development of SARS-CoV-2-related neurological injury, rather than direct viral invasion [37]. The authors investigated intracranial patterns of distribution of the particular neuropathologic findings as well. It appears that hypoxemic-ischemic lesions show a predilection for the cerebrum (86.2%), followed by cerebellar structures (49.3%), and brainstem (37.0%) [37].

There are several mechanisms that may explain the occurrence of cerebrovascular ischemic and hemorrhagic events in patients with COVID-19. To begin with, hypoxia per se promotes hypercoagulability, which leads to microthrombotic occlusion of the cerebral vessels and ischemic injury. Secondly, a SARS-CoV-2-related cytokine storm, and the resulting endothelial impairment, are also perceived as procoagulant factors [38].

In addition, histopathological evaluation of the brain specimen of COVID-19 patients with hypoxic-ischemic injury pointed to the presence of megakaryocytes in the cortical capillary bed–another possible source of the microvascular flow obstruction [37]. Interestingly, hypoxic-ischemic lesions were more frequent in COVID-19 patients with documented cardiovascular risk. This may imply that hypoxic-ischemic damage could be at least partially pre-existing in the investigated group of patients. On the contrary, certain cardiovascular conditions promote global hypoxemia and cerebral damage in COVID-19 patients [37,38].

Moreover, microvascular changes due to cardiovascular risk factors, such as arterial hypertension or diabetes mellitus, could foster the occurrence of hypoxic-ischemic lesions in COVID-19 patients. This latter hypothesis is in keeping with the evidence of an increased risk of vascular events in subjects with COVID-19, and with findings of a worse outcome of COVID-19 when premorbid vascular risk factors and diseases are present [37].

In their study, Thakur et al. performed neuropathological examinations of 20 to 30 areas in each from a total of 41 brains. Hypoxic-ischemic lesions, varying from acute to subacute, were detected in all the specimens. In the majority of cases, these were global or widespread injuries (32/41 patients, 78%). In five patients with diffuse cerebral edema and hypoxemic injury, the ischemic lesions were accompanied by concurrent or prior hemorrhagic foci. In nine individuals the hypoxic lesions were more focal, predominantly affecting the isocortex, hippocampi, brainstem, and/or cerebellum. In terms of histopathology, the acute ischemic injury presented as neuronal shrinkage and eosinophilia with or without neuronal loss, and reactive astrocytosis (confirmed by GFAP immunostaining). Subacute lesions comprised infarcts with variable macrophage infiltrates, reactive astrocytosis, and neovascularization. Robust accumulation of T lymphocytes was visible in either perivascular locations or within the brain parenchyma. Large vessel atherosclerosis and arteriosclerosis were seen in some individuals; however, there were no signs of vasculitis [35]. These findings advocate for no significant correlation between the histopathological alterations and levels of the virus in the brains of symptomatic individuals affected by COVID-19 [35].

Another systematic literature search (PROSPERO registration number: CRD42020221022) resulted in an analysis of 14 (out of 1608) articles on neuropathological findings in COVID-19-positive patients. Again, data indicated a relatively high incidence of hypoxic-ischemic changes (*n* = 41, 28.1%) in the studied patient sample; in several cases, these were accompanied by leptomeningeal inflammation (*n* = 7, 4.8%) [39].

In the study population, hypoxic-ischemic injury showed a predilection for the frontal cortex, optic chiasm, olfactory bulb, subcortical white matter, neostriatum, CA1 regions of hippocampi, and subtentorial structures (cerebellar Purkinje cells, in particular). Ischemic red neurons, pyknotic and eosinophilic neurons were detected in all the aforementioned hypoxia-sensitive regions, which is consistent with global hypoxic assault. In some cases, these were accompanied by microvascular lesions, i.e., microhemorrhages and microscopic ischemic foci, distributed within subcortical and deep white matter [39].

Bugra et al. reported the occurrence of acute hypoxic/ischemic changes in specimens from 54% of the analyzed study cohort (*n* = 54). However, the authors highlight the fact that, being largely non-specific, such lesions cannot be directly linked to COVID-19 [40].

Nauen and colleagues evaluated brain specimens from 15 individuals with nucleic acid-proven severe SARS-CoV-2 infection and confirmed pulmonary pathology. In five cases, large cell nuclei morphologically consistent with megakaryocytes were found in cortical capillaries; these were further confirmed by immunohistochemical staining–investigated cells were labeled with both CD61 and CD42b markers. Interestingly, such cells were not detected within cortical capillaries of the two COVID-19-negative patients with comparable age and hypoxic-ischemic lesions, chosen as a control group [41]. Seemingly megakaryocytic infiltration may be a unique pathological feature of the SARS-CoV-2 neuroinfection.

In terms of clinical features, hypoxic-ischemic lesions may result directly from COVID-19-related hypoxia with hypoperfusion, and/or as a complication of ventilation therapy or sedation. As the authors point out, the specific anatomical distribution of the foci, particularly in the callosal and capsular regions, were previously reported in cases of acute respiratory distress syndrome and extracorporeal membrane oxygenation (ECMO), which are not only associated with global hypoxia, but may also complicate the clinical course of severe SARS-CoV-2 infection (requiring invasive ventilation and pharmacotherapy, including sedatives and paralytics) [39].

### 3.2. Imaging Findings

El Beltagi et al. summarized imaging findings in hemodynamic hypoxic-ischemic events in the course of COVID-19, distinguishing three dominant patterns: watershed (arterial border zone) infarcts, hypoxic-ischemic encephalopathy–HIE (global hypoxic-ischemic injury) and delayed post-hypoxic leukoencephalopathy [42]. All the above-mentioned patterns tend to have different underlying pathophysiological mechanisms. Watershed infarcts most often are the effect of severe hypotension or significant arterial stenosis, and occur at the border between vascular territories, either in the cortical (external) zones or deep (internal) zones, parallel to the lateral ventricles [42,43,44]. HIE predominantly affects grey-matter structures with high metabolic demands, i.e., cerebral cortex, basal ganglia, thalami, and usually follows cardiac or respiratory arrest, or diffuse cerebrovascular disease [42,44,45,46]. On magnetic resonance (MR) images, global hypoxic-ischemic injury presents as areas of restricted diffusion within the basal ganglia, thalami, hippocampi, and cortex. Some reports indicated involvement and possible necrosis of the globus pallidus in HIE [45]. Delayed post-hypoxic leukoencephalopathy is a rare entity occurring after an acute hypoxic episode; secondary neurological decline observed 14 to 30 days after the primary injury, and following an apparent clinical improvement, seems to be closely related to the average turnover rate of myelin-related proteins [42,43,44,47] Prolonged hypoxemia leads to death of oligodendrocytes, the myelinating cells of CNS axons, thus resulting in demyelination of the cerebral white matter [44,47]. In addition, long-term hypoxia induces disruption of the blood–brain barrier, and thus capillary leakage, which may present as microbleed foci [43,44,47].

Thakur et al. reported a small series of five patients with diffuse cerebral edema and hypoxemic injury, three of whom also exhibited hemorrhages in imaging studies. Additional bilateral basal ganglionic petechial hemorrhagic lesions were reported in a single case [35]. Such observations are backed by numerous clinical series.

Sawlani et al. evaluated 3403 patients with confirmed severe acute respiratory syndrome due to SARS-CoV-2 infection. A total of 167 patients (4.9%) from the examined cohort presented neurological symptoms, prompting neuroimaging; these included delirium (26%), focal abnormalities (22%), and altered consciousness (20%). Abnormal MR or CT images were found in 23% of the examined patients (20 and 18 individuals). Microhemorrhagic foci (mostly within the splenium of corpus callosum) were the most common finding–60%, followed by acute/subacute infarcts (25%), and hypoxic-ischemic lesions within the basal ganglia (5%). Apart from the involvement of basal ganglia, bilateral hypoxic-ischemic lesions, manifesting as foci of restricted diffusion, were found within cerebral peduncles and tails of the hippocampi. Necrotic changes of the nigrostriatal tract were visible in some patients as well, and T2/FLAIR hyperintensities were detected within dentate nuclei and thalami [48].

A much smaller cohort of 47 patients with confirmed diagnosis (by SARS-CoV-2 RT-PCR) of COVID-19 pneumonia was evaluated by Mendez Elizondo et al. with 3T Siemens Magnetom Skyra setup. Only two patients exhibited abnormal MR findings; one within the olfactory bulbs, and one presenting lesions indicating hypoxic-ischemic encephalopathy [49].

Another retrospective observational study conducted in Pakistan included 12 patients with COVID-19 (positive polymerase chain reaction, PCR test) and neurological symptoms confirmed by neuroimaging. The most frequent indications for imaging studies in the study population were seizures and altered mental status.

Signs of hypoxic-ischemic encephalopathy were detected in three individuals (25%). One of the patients (a 35-year-old male with no comorbidities) suffered global ischemic encephalopathy, manifesting as an acute loss of consciousness. Another individual (a 41-year-old male with no comorbidities) was diagnosed with HIE with multifocal microhemorrhagic foci, presenting as drowsiness and altered mentation. The last one (a 56-year-old male with no comorbidities) experienced postictal, generalized tonic seizures due to global ischemic encephalopathy [50].

Radnis et al. published a small clinical series including three patients with hypoxic brain injury due to COVID-19 ARDS, not related to cardiopulmonary arrest. The first reported individual was a 32-year-old female with multiple comorbidities including asthma, obesity, gastroesophageal reflux, and vitamin D deficiency. She presented with deteriorating respiratory status (oxygen saturation below 85%, dyspnea), meeting the criteria of severe ARDS requiring intubation on the day of admission. Her clinical course was complicated by a cytokine storm with no episodes of prolonged hypotension. She was successfully extubated after 22 days of mechanical ventilation and presented signs of encephalopathy and right-sided facial twitching a single day afterwards. MR imaging indicated symmetric T2/FLAIR hyperintensities along with mild diffusion restriction within globus pallidi, posterior limbs of the internal capsules and both cerebral peduncles, which was consistent with hypoxic-ischemic injury. The second patient was a 42-year-old female with a history of obesity and a gunshot wound, who necessitated intubation a day after admission due to worsening hypoxemic respiratory failure not responding to a high-flow oxygen supply, and hypotension prompting cytokine release syndrome. After initial failure on day 16, she was successfully extubated after 22 days of mechanical ventilation. Five days post-extubation, she remained in a poor neurological state and an MRI demonstrated bilateral T2 hyperintensities within the subcortical white matter, perirolandic regions at the vertex, corona radiata, and centrum semiovale. The last, a 66-year-old male patient, presented with non-specific signs of cough, diarrhea, fever, and abdominal pain, and required intubation due to worsening hypoxemia (PaO2 of 61 on non-invasive ventilation). He experienced ventilation-related pneumonia and was finally extubated after a total of 44 days of mechanical ventilation. It was persistent encephalopathy that precluded earlier extubation, which manifested as bilateral, diffuse cortical, subcortical, thalamic, and cerebellar T2/FLAIR hyperintensities indicating hypoxemic-ischemic lesions. The authors conclude that the presence of imaging indicators for the hypoxic-ischemic injury in patients lacking severe hypoxemia may advocate for the role of hemoglobinopathy in the development of so-called “silent hypoxemia”, which is a state in which patients with COVID-19 pneumonia develop hypoxemia with mild or no clinical manifestations. It is likely that hypoxemia contributes to neurocognitive deterioration in patients with a severe clinical course of COVID-19, and a cytokine storm may be responsible for prolonged encephalopathy [51].

Sisniega et al. and Raghed et al. published single case reports of patients with apparent hypoxic-ischemic brain injury due to COVID-19 [44,52]. The first patient presented with scattered hyperintensities on T2/FLAIR and DWI MR images within cortical gray matter, caudate heads, and putamen, along with white matter–including symmetrical involvement of centrum semiovale and corona radiata. Areas of diffusion restriction did not correspond to any particular vascular territory [44]. The second described patient suffered from COVID-19-related multiple cardiopulmonary arrests, leading to global hypoxic-ischemic injury [52].

In addition, Fan et al. highlight that imaging features of encephalopathy in the course of COVID-19 may be accompanied by leptomeningeal enhancement and/or symmetric frontotemporal hypoperfusion [53].

## 4. Ischemic Stroke

A stroke is an acute medical condition characterized by a disruption of cerebral circulation. It can be classified into two main types: ischemic and hemorrhagic. Ischemic stroke can be caused by tissue hypoxia because of reduced blood supply, and this may be the result of thrombosis, thromboembolism, as well as systemic hypoperfusion.

Both may result in neurological disorders, such as inability to move or feel on one side of the body, difficulties in understanding or speaking, dizziness, or loss of vision on one side, regardless of which side the stroke occurred on.

When symptoms persist for less than 24 h, with no permanent injury to the cerebral nervous tissue, a transient ischemic attack (TIA), otherwise referred to as a mini-stroke, may be diagnosed. It has been noted that patients with COVID-19 infection may develop cerebrovascular disease (CVD), which manifests as either an acute or transient cerebrovascular accident [54].

### 4.1. Epidemiology

In May 2020, Ghannam et al. reported that cerebrovascular incidents account for 48.8% of all neurological events in COVID-19 patients; of these, 87.5% were ischemic strokes, 5% were cerebral venous thromboses, 5% were intraparenchymal hemorrhages, and 2.5% were subarachnoid hemorrhages [55]. The most frequent subtype of ischemic stroke was large vessel occlusion, which accounted for 77% of the 35 reported cases [27]. However, a retrospective cohort study of patients with ischemic stroke hospitalized within a major health system in New York, performed by Yaghi et al. indicated that large vessel disease contributed to only 6.2% out of a total of 32 ischemic stroke cases [56].

The prevalence of ischemic stroke among patients with COVID-19 is reported to be 1.6% by Klok et al., and 2.5% by Lodigiani et al., in studies from two separate institutes [12,57]. Therefore, the actual incidence of stroke in patients with COVID-19 may be even higher [58]. In support of these theoretical considerations, another study confirmed a significantly higher incidence of acute ischemic stroke in patients with COVID-19 infection when compared to those without [59].

A tool used by healthcare professionals with a high level of use in order to objectively determine the degree of impairment caused by a stroke is the NIH Stroke Scale (NIHSS).

Patients with COVID-19 and stroke have higher scores on the NIHSS compared to their counterparts without COVID-19 [56]. The mortality rate in acute ischemic stroke reaches 31%, while a pooled analysis revealed a dire 50% in COVID-19 patients complicated with acute ischemic stroke [60].

### 4.2. Risk Factors

Due to scientific curiosity as well as the need for further research, several theories are trying to relate inflammatory factors associated with SARS-CoV-2 infection to increased stroke risk. Researchers are making an effort to find any link between the cerebrovascular events and disruptions in metabolic pathways caused by COVID-19 infection, e.g., prothrombotic mechanisms and platelet aggregation, impaired lipid metabolism, alterations in endothelial function or instability of the atherosclerotic plaque with its subsequent rupture [61,62]. Patients with COVID-19 in general show increased levels of D-dimers, interleukin-6 (IL-6), C-reactive protein (CRP), fibrinogen, and platelets [63].

Substantial scientific evidence indicates that hypercoagulability, endothelial damage, and cardiogenic embolisms observed in severe clinical courses of the SARS-CoV-2 infection may all contribute to the occurrence of ischemic stroke [64]. However, the latest reports highlight that it is not only COVID-19 patients in severe clinical condition who may suffer from a stroke. A number of cases were published recently, in which a stroke was the primary/initial presenting symptom of the SARS-CoV-2 infection [65,66].

Hyperviscosity constitutes another major risk factor for thrombotic complications, which was demonstrated in a clinical series of critically ill patients with COVID-19. In the series, plasma viscosity was measured in 15 consecutive individuals (by means of traditional viscometry) revealing values above normal–ranging from 1.9 to 4.2 centipoise (normal range 1.4–1.8 centipoise) [67]. Nonetheless, hyperviscosity may well result from a substantial increase in fibrinogen concentrations, which is consistent with observations made in COVID-19 patients. Hyperviscosity promotes hypercoagulability by causing damage to endothelial cells [68]. Endotheliitis, which develops in response to direct viral invasion or systemic inflammation evoked by SARS-CoV-2, results in microvascular dysfunction and a hypercoagulable state, both increasing the risk of thrombotic events. Such endothelial dysfunction, together with anticoagulant treatment and certain comorbidities, may well promote the occurrence of a hemorrhagic stroke or hemorrhagic transformation of an ischemic stroke in COVID-19 patients [69,70].

### 4.3. Imaging Findings

Hernández-Fernández et al. described 17 cases of cerebral ischemia in COVID-19 patients. In the majority of cited cases (58.5% *n* = 10), large vessel occlusion was found on CT angiography (nine cases), or with transcranial ultrasound (one case). An extensive cortical infarction was found on the baseline head CT in three patients (presumably due to large vessel occlusion), which was not further evaluated by CT angiography (CTA). Another four patients failed to fulfill the criteria (M1, M2, intracranial carotid artery ICA, A1, P1, vertebral and basilar) of a stroke due to large vessel occlusion on CTA. Nonetheless, the authors did not record any COVID-19 patients with cerebral ischemia due to small vessel disease [71].

In a retrospective multicenter observational study, Mahammedi et al. reported a total of 34 acute ischemic stroke cases in 725 consecutive patients with concurrent COVID-19 infection. The majority of infarcts (19/34) were localized within large vascular territories: 15 in the middle cerebral artery, two in the posterior cerebral artery, and two in the anterior cerebral artery. The remaining cases were classified as: small infarcts (*n* = 11), cardioembolic (*n* = 3), and hypoxic-ischemic encephalopathy pattern (*n* = 1) [72].

In a case series, Beyrouti et al. described six patients with ischemic stroke due to large vessel occlusion in the course of SARS-CoV-2 infection. In three cases, the infarct area affected more than one vascular territory. Of these, one patient presented with acute left vertebral artery thrombus and acute left posterior-inferior cerebellar artery infarction, another with acute right parietal cortical and left cerebellar infarct, whereas the remaining one had multiple infarct zones within the right thalamus, left pons, right occipital lobe, and right cerebellar hemisphere due to occlusion of the basilary artery with bilateral mild-to-moderate P2 segment stenosis. Interestingly, in two out of six reported cases, ischemic stroke occurred despite anticoagulant treatment. In another two cases, concurrent venous thrombosis was observed [73].

### 4.4. Conclusions

Given all of the above, COVID-19 patients with ischemic stroke should undergo a complete panel of diagnostic tests (including brain imaging, vascular imaging, and cardiac evaluation) and laboratory tests (CRP, D-dimer, fibrinogen and cytokines) [74].

According to the international guidelines for the early management of patients with acute ischemic stroke (2019 update), intravenous thrombolysis with rtPA can be recommended in selected patients within 3 h, and highly selected patients within 3 to 4.5 h from symptom onset. Mechanical thrombectomy is recommended for large vessel occlusion within six hours of symptom onset. This recommendation comes with the caveat that patients with COVID-19 infection tend to have elevated plasma concentrations of the inflammatory and hypercoagulability markers, which was associated with an increased risk of death or disability, and post-thrombolytic intracranial hemorrhage, as compared to individuals without concurrent COVID-19 infection [74,75].

Lately, a new approach to ischemic stroke treatment in COVID-19 patients has emerged as a sequela of the postulated affinity of the virus to ACE2. This includes exogenous supplementation of ACE2 by administering human recombinant soluble ACE2 (hrsACE2), which proved not only to inhibit SARS-CoV-2 infection in a laboratory model, but also to impede the COVID-19-related ACE2 depletion, and thus prevent endothelial dysfunction. Other treatments targeting the renin–angiotensin system are also under investigation as a promising preventive therapy against stroke in SARS-CoV-2 infection [76].

## 5. Hemorrhagic Stroke

### 5.1. Epidemiology

As compared to ischemic events, fewer cases of hemorrhagic strokes are observed in patients with COVID-19 infection [4]. Based on a cohort of 755 COVID-19-positive patients (out of a total of 3824) in whom neuroimaging studies were performed during hospitalization, Dogra et al. estimated that the prevalence of intracranial hemorrhage (ICH) to be 4.4% (*n* = 37), which accounted for nearly 11% of all strokes in the investigated group. Almost 80% of the ICHs occurred in male patients. The mean age of COVID-19 patients who suffered from acute cerebrovascular disease was higher compared to unaffected individuals with infection. 15% of ICHs were parenchymal hemorrhages with mass effect and herniation, with the mortality rate reaching 100% [29]. Punctate hemorrhages accounted for 25% of ICHs, whereas small-to-moderate hemorrhages were observed in 60% of patients. Noteworthy, 67% of the investigated individuals received therapeutic, and 9% prophylactic, anticoagulation prior to the onset of ICH [4,27,29,30,77,78].

In a systematic review, Maury et al. analyzed data regarding COVID-19-related ICHs from nine cohort studies from Belgium, China, France, Italy, the United Kingdom, and the US [79]. The estimated prevalence of ICH oscillated between 0.2% in the French cohort, which included 46 centers (Meppiel et al.) [79,80], and 0.8% in the three-center study from Italy (Mahammedi et al.) [72,79].

In a retrospective study, Ramos et al. compared 84 patients with radiologically diagnosed acute stroke from 2020 (pandemic), with 152 individuals from the historical cohort. As compared to the historical and 2020 COVID-negative cohorts, infected patients were more likely to experience a hemorrhagic conversion of the ischemic stroke, while no difference was observed between the groups regarding the incidence of ICH. In addition, study results indicated higher ICH rates among COVID-positive patients receiving anticoagulation, as opposed to COVID-negative subjects [81]. Topcuoglu et al., in a prospective case-control study, described two cases of acute ICH and COVID-positive out of a total of 46 patients with ICH included in the cohort. COVID-positive patients in this study were characterized by a higher NIHHS score on admission, were younger (mean age 54 vs. 67 years) and had a higher mortality rate (50% vs. 30%). In one of the presented cases, ICH presumably occurred due to warfarin overdose; also, a single case of hypertensive ICH was noted [82].

### 5.2. Risk Factors

Hypertension is the most important risk factor for cerebral hemorrhage [4]. Reported prevalence of hypertension was between 14% (non-critical COVID-19 patients) to 23,7% (critical COVID-19 patients) [5]. Reduced angiotensin-converting enzyme 2 (ACE2) expression contributes to an increased level of angiotensin 2 in serum and, in patients with pre-existing hypertension, may lead to large blood pressure fluctuations.

Type 2 diabetes mellitus is also an independent risk factor for hemorrhagic stroke, as well as elevated plasma D-dimer levels [3,6,27,83]. Smoking is another independent major risk factor for hemorrhagic stroke, especially in critical COVID-19 patients [3,6,27]. Research has shown that excessive adrenergic stimulation by catecholamines during increased anxiety, fear, and stress could also lead to severe vasospasm and microcirculation disturbances, which increase the risk of hemorrhagic stroke. The use of ECMO is an indirect risk factor in patients who develop severe acute respiratory distress syndrome due to the administration of anticoagulants [67,68].

### 5.3. Imaging Findings

In a case series study, Benger et al. presented clinical features including imaging findings of five cases of COVID-19 associated ICH. Two cases demonstrated acute intraparenchymal hematoma in the left and right frontoparietal lobe, respectively; in one case subacute intraparenchymal hematoma in the right superior frontal gyrus was discovered in CT. In the last two cases, ICH localized, respectively, in the right basal ganglia and multilobar right perirolandic, left frontal, and left cingulate. In CT angiography, none of the patients had vascular abnormality underlying ICH. There were also no imaging markers of hemorrhagic conversions of infarcts in this study [22,23].

Nawabi et al. described 18 cases of ICH in the course of SARS-CoV-2 infections. Most of the described cases (72.2%, *n* = 13) concerned subarachnoid hemorrhage, 33.3% (*n* = 6) parenchymal, 16.7% (*n* = 3) intraventricular, and one was subdural hemorrhage (5.6%, *n* = 1). One of the patients, besides ICH, presented disseminated microbleeds on MRI susceptibility-weighted images, which the authors linked with acute respiratory distress syndrome. In this study, eight out of 18 patients showed no neurovascular pathology in CT angiography. The authors also excluded hemorrhagic conversion of ischemic stroke from the study [23,84].

In the narrative review, Margos et al. analyzed a few other reports concluding that, out of a total of 114 cases of ICH with reported location, most of them (*n* = 64, 56%) were located at lobar regions, seven at basal ganglia, 13 were supratentorial, 13 infratentorial, seven non-lobar and 10 were localized multifocal. In the same review, authors presented that 149 (79%) of 188 total ICHs cases were primary, including cases with all known ICH risk factors (e.g., use of anticoagulants), 29 ICHs were secondary to hemorrhagic conversion of ischemic stroke, seven occurred due to vein thrombosis, and three to encephalopathy [23].

### 5.4. Indirect Links between COVID-19 and Hemorrhagic Stroke

Apart from the direct damage caused by SARS-CoV-2 viral infection, the COVID-19 pandemic indirectly influenced the outcomes of patients with hemorrhagic stroke (HS) in a number of different ways. In a retrospective study, Chen et al. compared 224 patients who suffered from HS during the pre-COVID era, with 126 individuals from the pandemic period. The authors concluded that clinical outcomes of patients with moderate HS (NIHSS score between 5 and 15) during the pandemic were relatively worse in comparison with patients from the pre-COVID period (26.3% vs. 53.1% good outcomes) [85,86]. One of the possible explanations is that such differences may be caused by the prolongation of door-to-department time by approximately 50 min during the pandemic, compared to the pre-COVID-19 period.

In addition, higher rates of pneumonia complications were also reported in patients with hemorrhagic stroke during the pandemic, as opposed to the pre-COVID-19 period (60.7% vs. 40.6%) [85].

The only currently available treatment is to prevent COVID-19 and/or reduce the severity of COVID-19 symptoms through vaccination. Nonetheless, there are several preliminary case reports in which HS often coexists with vaccine-induced thrombotic immune thrombocytopenia (VITT) and cerebral venous sinus thrombosis (CVST) within 2–8 days after the first or second dose of COVID-19 vaccine [45,46,47]. In the majority of cases, previously healthy patients presented with thrombocytopenia, elevated D-dimer and low fibrinogen levels, and often high concentrations of the anti-PF4 (platelet factor 4) antibodies [13,87,88]. Since VITT and CVST are both reported as very rare adverse reactions to COVID-19 viral vector vaccines, most reports relate to the ChAdOx1 nCoV-19 vaccine (AstraZeneca). However, there were also reports describing similar symptoms after Janssen (Ad26.COV2.S) [89] as well as Comirnaty mRNA (Pfizer-BioNtech) and mRNA-1273 (Moderna) vaccines [90,91,92].

## 6. Conclusions

Cardiovascular disease is present in a large number of patients affected by COVID-19. A meta-analysis of six published studies from China involving 1527 patients with COVID-19 reported common comorbidities, including diabetes (9.7%), cardiovascular disease (16.4%), and hypertension (17.1%) [93]. Nearly half of the neurological events in COVID-19 include cerebrovascular incidents, which, based on a summary of the 2018 China Cardiovascular Disease Report, resulted in more than 13 million patients [94]. As for the disease entities discussed in this review, the most common of these was ischemic stroke, while the second was intracranial hemorrhage [55]. Scientific articles provide a direct link between nasopharyngeal viremia and hypoxemia [95]. Table 1 summarizes and compares the epidemiology, neuropathology, and risk factors of the conditions discussed.

On diagnostic imaging, hypoxic-ischemic events may present as watershed infarcts, hypoxic-ischemic encephalopathy (HIE), and delayed post-hypoxic leukoencephalopathy [42]. Such lesions appear on MR images as areas of restricted diffusion (bright DWI and low ADC signal) involving the basal ganglia, thalami, hippocampi, and cerebral cortex.

Hypercoagulability occurs as a sequela of hypoxia, resulting in microthrombotic occlusion of the cerebral vessels and, as a consequence, ischemic cerebral injury. MRI signal patterns of acute ischemic stroke are similar to hypoxic-ischemic lesions, which may indicate the continuum between the small hypoxic area and a well-defined infarction territory. However, the location of the infarct zones corresponds to/is delineated by particular vascular territories.

Recent literature evidence indicates that both the hemorrhagic and ischemic cerebral infarction in the course of COVID-19 may have the same underlying pathomechanism. The authors postulate that virus affinity to ACE2 receptors is the key point for cerebrovascular disease to occur in SARS-CoV-2 infection. The interactions between SARS-CoV-2 and ACE2 seem to result in endothelial and cerebral vascular damage, and disintegration of the blood–brain barrier. However, the molecular mechanism has not been fully elucidated yet, and requires further investigation [4,5,6,7].

According to other possible hypotheses, hypoxemia contributes to the activation of several hypoxia-inducible factors (HIF-2α), also known for inducing or inhibiting several important genes involved in normal hemostatic mechanisms [96].

Another possible explanation refers to the effects of hypoxemia, and hypoxia-inducible factors (HIF-2α), which have a regulatory function over several genes involved in normal homeostatic mechanisms [96].

In terms of clinical practice, evaluating the effects of COVID-19 infection on cerebral circulation is a fundamental issue for neurologists, whereas neuroradiologists aim to identify the imaging indices of the microscopic brain damage caused by viral infection and its sequelae. The previous section presented all the MRI findings that were described, and should alert radiologists to the possibility of subacute ischemic lesions typical for SARS-CoV-2 patients.

Such lesions present a different image than cortical ischemia, and it was also observed that they may resolve spontaneously in subsequent MR studies. However, such normalization of MRI images may be the result of potentially reversible alterations of the cerebral microcirculatory function. Cortical dysfunction and ischemia are probably caused by disrupted local homeostasis or extracellular contents triggered by changes in the blood–brain barrier [97]. The ischemic events in COVID-19 present different imaging patterns than cortical ischemia and, surprisingly, they may spontaneously resolve in subsequent MRI studies. Such normalization of the MRI signal may represent reversible alterations, or temporary dysregulation, of the cerebral microvascular function. Cortical dysfunction and ischemia possibly occur due to impairment of the local homeostasis, or as a result of the accumulation of extracellular contents triggered by disruption in the blood–brain barrier [97].

Furthermore, it is worth mentioning that several authors reported the possible occurrence of cerebrovascular events (both ischemic and hemorrhagic) following the administration of the COVID-19 vaccine. In the majority of reported cases, patients received vector-type vaccinations (mainly Astra-Zeneca). However, a small number of case reports described similar adverse effects after mRNA vaccines. While it has been postulated that post-vaccine ICH occurs mainly due to hemorrhagic transformation of cerebral venous thrombosis caused by autoimmune thrombocytopenia (resembling heparin-induced thrombocytopenia), the exact mechanism is still not fully understood and requires further elucidation [90,91,92].

Despite being comprehensive in nature, the review includes limitations that should be noted by the reader. It is important to underline that, since the beginning of the COVID-19 pandemic in 2019, we have experienced consecutive infection waves caused by successive viral mutations. The viral strains or variants differed in their virulence toward particular body systems, and as the worldwide observations suggest, were associated with higher or lower cerebrovascular event rates. Nevertheless, until now, there is very little scientific evidence to support such a general or common sense assumption, so one should keep in mind the possibility that future viral mutants may present different clinical behavior. This leads us directly to the second relevant aspect. Although more and more scientific reports are available regarding possible molecular mechanisms of SARS-CoV-2 cerebral vascular damage, the pathological key elements of neuroinfection are yet to be elucidated before they can be translated to everyday clinical work e.g., as prognostic markers.

## Figures and Tables

**Table 1:** Epidemiology, localization, and risk factors of the discussed cerebrovascular conditions in COVID-19 patients.

Parameter/Condition	Hypoxic-Ischemic Encephalopathy	Acute Intracranial Hemorrhage	Acute Ischemic Stroke
**Prevalence**	28–50%, some authors concluded that hypoxic-ischemic lesions are non-specific and cannot be directly linked to COVID-19	0.2–0.8%	1.6–2.5%
**Mortality rate**	n/a	~35–50%	~19.1–31%
**Neuro-pathology localization**	various: frontal cortex, optic chiasm, olfactory bulb, subcortical white matter, neostriatum, CA1 regions of hippocampi, subtentorial structures	most commonly lobar (15–30%), multilobar, basal ganglia	large vessel occlusion, most common: middle cerebral artery (>50%),
**Independent risk factors**	ARDS, use of ECMO	hypertension, type 2 diabetes mellitus, elevated plasma D-dimer levels, smoking, use of ECMO	hyperviscosity, use of ECMO, endotheliitis and other endothelial dysfunction, elevated plasma D-dimer levels, dyslipidemia, atrial fibrillation

## Data Availability

Not applicable.
